# Immune Checkpoint Inhibitors in the Treatment of Patients With Cancer and Preexisting Psoriasis: A Systematic Review and Meta-Analysis of Observational Studies

**DOI:** 10.3389/fonc.2022.934093

**Published:** 2022-07-15

**Authors:** Yixuan Yu, Yang Zhou, Xu Zhang, Kexin Tan, Jiabin Zheng, Jia Li, Huijuan Cui

**Affiliations:** ^1^ Graduate School, Beijing University of Chinese Medicine, Beijing, China; ^2^ Oncology Department of Integrative Medicine, China-Japan Friendship Hospital, Beijing, China

**Keywords:** immune checkpoint inhibitor (ICI), immune-related adverse event (irAE), psoriasis, autoimmune disease (AID), programmed cell death 1 (PD-1), programmed cell death ligand 1 (PD-L1), cytotoxic T lymphocyte-associated protein 4 (CTLA-4)

## Abstract

**Background:**

Immunotherapies represented by immune checkpoint inhibitors (ICIs) have revolutionized cancer treatment. A large part of the population has both cancer and psoriasis but is usually excluded from ICI clinical trials because of the dysregulated activation of the immune system. This is the first study to evaluate the safety and efficacy of ICI therapy in patients with cancer and preexisting psoriasis.

**Methods:**

PubMed, EMBASE, Cochrane, and MEDLINE databases were searched from inception through February 2022. Observational studies on patients with cancer and confirmed psoriasis before ICI initiation were included. Outcomes included the incidence of psoriasis flares, *de novo* immune-related adverse events (irAEs), discontinuation rate due to flare/*de novo* irAEs, and efficacy of ICI therapy. Clinical manifestations, management, and outcomes for adverse events (AEs) were systematically reviewed. All pooled analyses were based on a random-effects model using Stata software. Meta-regression and subgroup analyses were performed to identify sources of heterogeneity.

**Results:**

Twelve studies involving 191 patients were included. The pooled incidence of psoriasis flares was 45.0% (95% CI: 31.1%-58.9%, I^2^ = 71.7%) and 44.9% (95% CI: 29.0%–60.7%, I^2^ = 71.8%) for *de novo* irAEs. The tumor type, psoriasis subtype, ICI class, and country were the main sources of heterogeneity. Grade 3–4 flares occurred in 10.8% (95% CI: 5.3%–16.3%) of patients, and about 16.6% (95% CI: 10.7%–22.5%) of patients experienced grade 3–4 *de novo* irAEs. The estimated incidence of ICI discontinuation due to AE was 18.5% (95% CI: 6.1%–30.8%, I^2^ = 68.7%). The median times to develop flare and *de novo* irAEs were 44 and 63 days, respectively. Endocrinopathies and colitis were the most common *de novo* irAEs. Conventional therapy is effective for most AEs. The estimated objective response rate (ORR) of ICIs was 38.1% (95% CI: 11.8%–64.3%, I^2^ = 81.7%), and the disease control rate (DCR) was 64.5% (95% CI: 55.3%–73.8%, I^2^ = 0).

**Conclusions:**

The flare of patients with cancer and preexisting psoriasis treated with ICI therapy is frequent, but the incidence of *de novo* irAEs and the efficacy of ICI therapy are comparable to those of the general population. Most AEs are mild and manageable with conventional therapy, which required discontinuation of ICI therapy in 18.5%.

**Systematic Review Registration:**

https://www.crd.york.ac.uk/prospero/, identifier CRD42022320646

## Introduction

Cancer is the second leading cause of death in the United States, with 608,570 cancer-related deaths reported in 2021 (1). Recently, immunotherapies represented by immune checkpoint inhibitors (ICIs) have revolutionized the management of cancer. Nevertheless, the prevalence of immune-related adverse events (irAEs) and their clinical efficacy remain alarming.

Psoriasis is a common autoimmune disease (AID) characterized by chronic T cell-mediated inflammation. It predominantly affects the skin (psoriasis) and joints [psoriatic arthritis (PsA)]. Psoriasis lesions manifest mainly as plaques, whereas other manifestations include guttate, flexural, erythrodermic, pustular palmoplantar, nail psoriasis, and arthritis of PsA (2). Notably, a significant number of patients suffer from both cancer and psoriasis. In a large register-based analysis of 2,10,509 lung cancer patients, 2.8% of the patients also had comorbid psoriasis (3). In a recent meta-analysis (4), the prevalence of cancer in patients with psoriasis was 4.78% (95% CI: 4.02%-5.59%), which was not trivial.

Blocking antibodies against programmed cell death receptor-1 (PD-1), programmed cell death ligand-1 (PD-L1), and cytotoxic T lymphocyte-associated molecule-4 (CTLA-4) checkpoint molecules are the most widely used type of ICIs (5). In the past, ICI therapy was thought to trigger severe autoimmune manifestations. This concern stems from the fact that both checkpoint molecules contribute to self-tolerance maintenance. CTLA-4 inhibitors interrupt the interaction of CTLA-4 with B7 co-stimulatory molecules and decrease regulatory T cell (Treg)-suppressive function, while PD-1/PD-L1 inhibitors block the PD-1/PD-L1 pathway (6, 7). Both can restore T-cell activation. ICIs can also activate helper T cells 1 and 17, which produce interleukin-17 (IL-17) and play a crucial role in the pathogenesis of various AIDs (8). Moreover, previous immunosuppressive treatments are thought to compromise ICI efficacy (9).

Hence, patients with preexisting psoriasis were always excluded from the initial clinical trials. However, the latest National Comprehensive Cancer Network (NCCN) guidelines demonstrated that these patients are still potential users of ICIs. More trials recently have included patients who do not require systemic therapy (10). Because of the few studies that have reported patients with both cancer and preexisting AIDs covering a small sample of psoriasis patients, fewer details of ICI therapy in patients with psoriasis are known. Hitherto, the safety and efficacy of ICI therapy in this population are still inconclusive.

To provide evidence for clinical decisions, this study aimed to estimate the prevalence of adverse events (AEs) and the response rate of ICI therapy and to review the clinical manifestations, management, and outcomes of these AEs in patients with cancer and preexisting psoriasis.

## Methods

### Protocol and Registration

This study was conducted according to the Preferred Reporting Items for Systematic Reviews and Meta-Analyses (PRISMA) statement, and the protocol has been registered with the International Prospective Register of Systematic Reviews (PROSPERO) (registration number: CRD42022320646).

### Literature Search and Eligibility Criteria

We searched PubMed, EMBASE, Cochrane library databases, and MEDLINE without language restriction from database inception through February 9, 2022.

The search terms mainly included cancer, tumor, immune checkpoint inhibitor, psoriasis, and pre-existing. The specific electronic search strategy is provided in **Supplementary Material 1**.

The inclusion criteria were observational studies (cohort studies, case–control studies, or case series, prospective or retrospective, excluding single case reports) that reported the incidence of irAEs or the efficacy of ICI therapy. The study population included patients with cancer and a confirmed diagnosis of psoriasis (including PsA and other subtypes) before ICI therapy (anti-PD-1/anti-PD-L1/anti-CTLA-4 or combination). The outcomes of patients with psoriasis were reported separately.

We excluded a) studies that only included patients with pre-immunotherapy-induced psoriasis, b) the treatment regimen involved therapies other than ICI (such as targeted therapy, chemotherapy, radiation therapy, or other immunotherapies), c) duplicated studies, and d) studies in which the full text cannot be obtained.

### Study Selection and Data Extraction

Two reviewers (YY and YZ) independently participated in the primary screening of the titles and abstracts, full-text reading, and final decisions. Discrepancies and disagreements were resolved through discussion. If the disagreement could not be resolved, a third-party (HC) expert was consulted.

The data were extracted independently by four reviewers (YY, YZ, XZ, and KT) using the pretested electronic form (**Supplementary Table 1**) and then cross-checked. The primary outcomes included the incidence of psoriasis flares, *de novo* irAEs, discontinuation due to flare/*de novo* irAEs, and efficacy of ICI therapy. Efficacy was measured using objective response rate (ORR) and disease control rate (DCR) [complete response (CR), partial response (PR), and stable disease (SD); ORR, CR + PR; DCR, CR + PR + SD].

### The Risk of Bias Assessment and Publication Bias

The Strengthening the Reporting of Observational Studies in Epidemiology (STROBE) instrument was used to assess the risk of bias, as no appropriate assessment tool was applied. STROBE consists of 22 items to evaluate observational studies. Each item would be labeled as “Yes” or “No,” and total fractions were expressed as a percentage of this value. The risk of bias was scored as follows: 0%–25%, high risk; 25%–50%, medium to high risk; 50%–75%, low to medium risk; and 75%–100%, low risk. Eligibility was evaluated by two reviewers (KT and JL), and disagreements were resolved by a third reviewer (HC). The possibility of publication bias was assessed using Egger’s test, which can be seen in funnel plots.

### Data Synthesis and Analysis

To start with, meta-analyses were performed by calculating the incidence and response rate with 95% CI based on the random-effects model according to heterogeneity.

Heterogeneity was assessed using the I^2^ statistic. When I^2^ was greater than 50%, a meta-regression analysis was performed to identify the sources of heterogeneity. Second, subgroup analysis was carried out according to the types of cancer, class of ICIs, psoriasis subtypes, etc. For comparing differences in the incidence between the subgroups, we used the chi-square test, continuity correction chi-square test, or Fisher’s exact test.

The data were recorded in Microsoft Excel and analyzed using Stata 15.0. Statistical significance was defined as a two-sided p-value of <0.05.

## Results

### Study Selection and Characteristics

**Figure 1:**
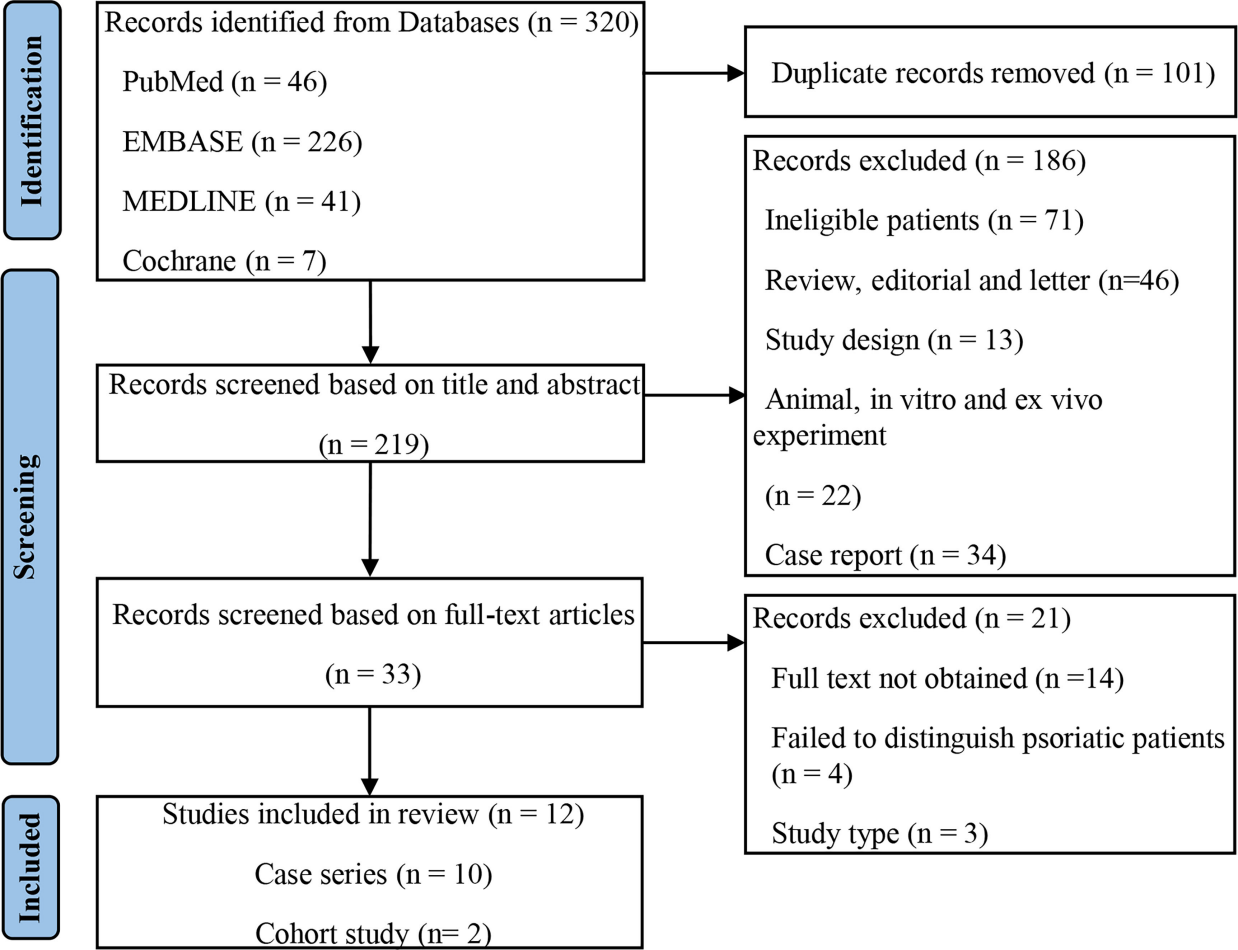
Search and selection flow diagram.

A total of 320 records were retrieved from the primary search, and 219 remained after adjusting for duplicates. Screening and eligibility assessment of the titles and abstracts was performed, and 33 studies were included. Finally, 12 studies (11–22) met the eligibility criteria, and the selection flowchart is shown in **Figure 1**.

All studies (11–22) were published in English. The publication dates ranged from 2016 to 2021. Two studies (15, 18) were prospective. Eleven studies (11–14, 16–22) were multicenter, and the majority of these studies were conducted in the United States, Australia, France, Germany, and Spain. A total of 191 participants were included, with four studies (11, 18, 19, 21) including patients with psoriasis, whereas the remaining eight studies (12–17, 20, 22) included patients with psoriasis and PsA. One study (20) targeted psoriatic patients only, whereas 11 studies (11–19, 21, 22) included participants with cancer and any AIDs. Five studies (11–14, 19) included patients with melanoma, two studies (16, 22) included patients with non-small cell cancer (NSCLC), one study (18) focused on urologic cancer, and the remaining (11–15, 17, 19–21) were mixed. The classes of ICIs included the following: anti-PD-1 (12, 13, 15, 17, 22), anti-PD-L1 (17, 18) anti-CTLA-4 (11, 14), and combination (16, 19–21). All of these studies have reported the safety and efficacy of ICI therapy. The median follow-up ranged from 4.7 to 25.1 months. The study characteristics are described in **Table 1**, and more details can be found in **Supplementary Table 2**.

**Table 1:** Basic characteristics of the included studies.

Author-year	Patients (n)	Psoriasis clinical subtype	Type of cancer	ICI class	Flare, n (%)	G3–4, n (%)	*De novo* irAEs, n (%)	G3–4, n (%)	Discontinuation, n (%)	ORR (%)	DCR (%)
Johnson-2016 (11)	5	Psoriasis	Melanoma	Anti-CTLA-4	1 (20.0%)	NA	4 (80.0%)	1 (20.0%)	1 (20.0%)	100.0%	100%
Menzies-2017 (12)	8	Psoriasis and PsA	Melanoma	Anti-PD-1	4 (50.0%)	NA	NA	NA	NA	NA	NA
Gutzmer-2017 (13)	3	Psoriasis vulgaris	Melanoma	Anti-PD-1	1 (33.3%)	0	0	0	0	0	0
Kähler-2018 (14)	7	Psoriasis and PsA	Melanoma	Anti-CTLA-4	2 (28.6%)	NA	2 (28.6%)	NA	NA	28.6%	42.9%
Danlos-2018 (15)	12	Psoriasis and PsA	Melanoma, NSCLC, etc.	Anti-PD-1	4 (33.3%)	NA	NA	NA	NA	NA	NA
Leonardi-2018 (16)	14	Psoriasis and PsA	NSCLC	Anti-PD-1 or anti-PD-L1	5 (35.7%)	0	7 (50.0%)	1 (7.1%)	1 (7.1%)	10.0%	60.0%
Tison-2019 (17)	31	Psoriasis and PsA	Melanoma, NSCLC and urologic cancer	Anti-CTLA-4, anti-PD-1, or combination	21 (67.7%)	4 (12.9%)	13 (41.9%)	5 (16.1%)	6 (19.4%)	NA	NA
Loriot-2020 (18)	15	Psoriasis	Urinary tract carcinoma	Anti-PD-L1	2 (13.3%)	0	2 (13.3%)	2 (13.3%)	1 (6.7%)	NA	NA
Brown-2021 (19)	6	Psoriasis	Melanoma	Combination of anti-CTLA-4 and anti-PD-1	4 (66.7%)	1 (16.7%)	NA	NA	NA	66.7%	83.3%
Halle-2021 (20)	76	Psoriasis vulgaris, pustular and PsA	Melanoma, NSCLC, head and neck, EAC, etc.	Anti-CTLA-4, anti-PD-1, anti-PD-L1 or combination	43 (56.6%)	7 (9.2%)	45 (59.2%)	17 (22.4%)	27 (35.5%)	Melanoma, 57.7%; total, 52.1%	Melanoma, 65.3%; total, 65.2%
Hoa-2021 (21)	7	Psoriasis and PsA	Melanoma, lung cancer, etc.	Anti-PD-1 or combination of anti-PD-1 and anti-CTLA-4	6 (85.7%)	2 (28.6%)	4 (57.1%)	1 (14.3%)	0	NA	57.1%
Calvo-2021 (22)	3	Psoriasis	NSCLC	Anti-PD-1	1 (33.3%)	NA	1 (33.3%)	NA	1 (33.3%)	NA	NA

ICI, immune checkpoint inhibitor; G3–4, grades 3–4; ORR, objective response rate; DCR, disease control rate; CTLA-4, cytotoxic T lymphocyte-associated protein 4; PD-1, programmed cell death 1; PD-L1, programmed cell death ligand 1; PsA, psoriatic arthritis; irAEs, immune-related adverse events; NSCLC, non-small cell lung cancer; EAC, esophageal adenocarcinoma; NA, not available.

### Psoriasis Flare, Clinical Manifestation, and Management

**Figure 2:**
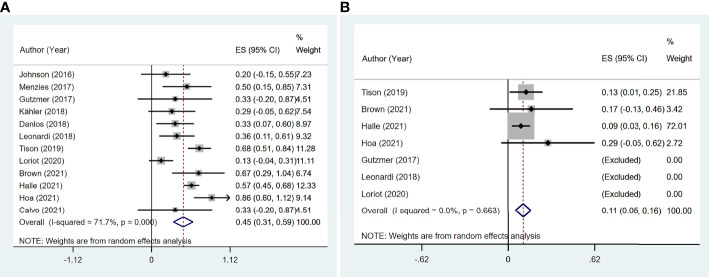
The pooled incidence of flare in patients with cancer and preexisting psoriasis. **(A)** The incidence of flare for any grades. **(B)** The incidence of flare for grades 3–4.

A flare was defined as a recurrent or worsening of prior psoriasis syndrome, which was reported in all 12 studies (11–22), involving 191 participants. The pooled meta-analysis showed that the incidence of flares was 45.0% (95% CI: 31.1%–58.9%) based on a random-effects model with significant heterogeneity (I^2^ = 71.7%, p < 0.001; **Figure 2A**).

In the meta-regression, the study design showed statistical significance (p = 0.027, 95% CI: −57.8%–3.5%; **Supplementary Table 3**).

Further subgroup analysis revealed heterogeneity in the mixed tumor type (I^2^ = 66.6%, p = 0.032) and mixed psoriasis subtype (I^2^ = 60.0%, p = 0.020). The incidence of flare was 39.5% (95% CI: 22.8%–56.2%) in melanoma and 35.3% (95% CI: 12.6%–58.0%) in NSCLC in the subgroup analysis based on cancer types (**Supplementary Figure 1**).

**Figure 3:**
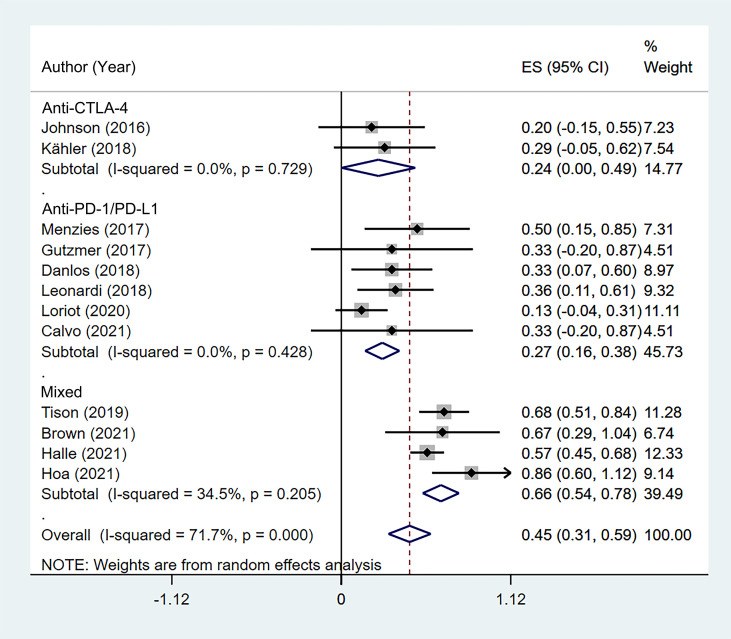
Subgroup analysis of the pooled incidence of flare in patients with cancer and preexisting psoriasis based on the class of immune checkpoint inhibitors (ICIs).

According to the class of ICIs, the incidence of flares was 24.5% (95% CI: 0.3%–48.7%) for anti-CTLA-4, 27.0% (95% CI: 15.8%–38.3%) for anti-PD-1/PD-L1, and 65.6% (95% CI: 53.7%–77.5%) for the mixed group (**Figure 3**). There was no difference in incidence between the anti-CTLA-4 group and anti-PD-1/PD-L1 group χ^2^ = 0.03, p = 0.954).

In terms of clinical manifestations, 12 patients (11, 14, 16, 21) and a 76-scale case series (20) of flare onset time were reported, with a median time of 44 days, ranging from 4 to 725 days. The pooled incidence of grade 3–4 flares was 10.8% (95% CI: 5.3%–16.3%, I^2^ = 0, p = 0.663; **Figure 2B**).

Topical steroids and vitamin D analogs (including calcipotriol and calcitriol) were used to treat the majority of the patients. Eighty patients received phototherapy, six patients used acitretin additively, and a few patients with severe flare syndrome were given an oral steroid, methotrexate, apremilast, or biologic agents in addition. The majority of the flares were controlled.

### *De novo* Immune-Related Adverse Event, Clinical Manifestation, and Management

**Figure 4:**
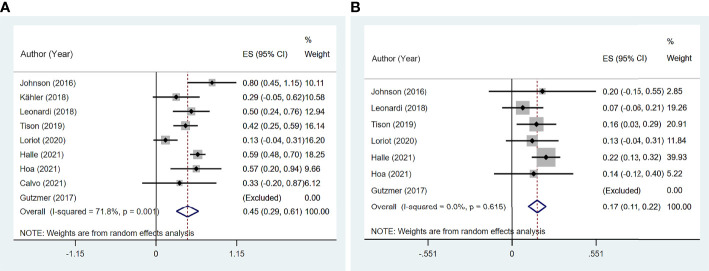
The pooled incidence of *de novo* immune-related adverse event (irAE) in patients with cancer and preexisting psoriasis. **(A)** The incidence of flare for any grades. **(B)** The incidence of *de novo* irAE for grades 3–4.

The incidence of *de novo* irAEs was available for nine studies (11, 13, 14, 16–18, 20–22), involving 161 participants. The incidence of *de novo* irAEs varied from 0% to 80.0%, with a pooled result of 44.9% (95% CI: 29.0%–60.7%). However, significant heterogeneity was found between studies (I^2^ = 71.8%, p = 0.001; **Figure 4A**).

Meta-regression showed significant heterogeneity between US studies and other studies (p = 0.010, 95% CI: 8.8%–65.4%; **Supplementary Table 4**).

Significant heterogeneity was found in the psoriasis (excluded PsA) group (I^2^ = 82.2%, p = 0.004), melanoma group (I^2^ = 76.9%, p = 0.038), anti-CTLA-4 group (I^2^ = 76.9%, p = 0.038), and anti-PD-1/PD-L1 group (I^2^ = 62.7%, p = 0.069) based on subgroup analysis. In addition, subgroup analysis indicated that the incidence of *de novo* irAEs was 54.0% (95% CI: 3.6%–104.4%) in melanoma and 46.8% (95% CI: 23.3%–70.3%) in NSCLC (**Supplementary Figure 2**).

**Figure 5:**
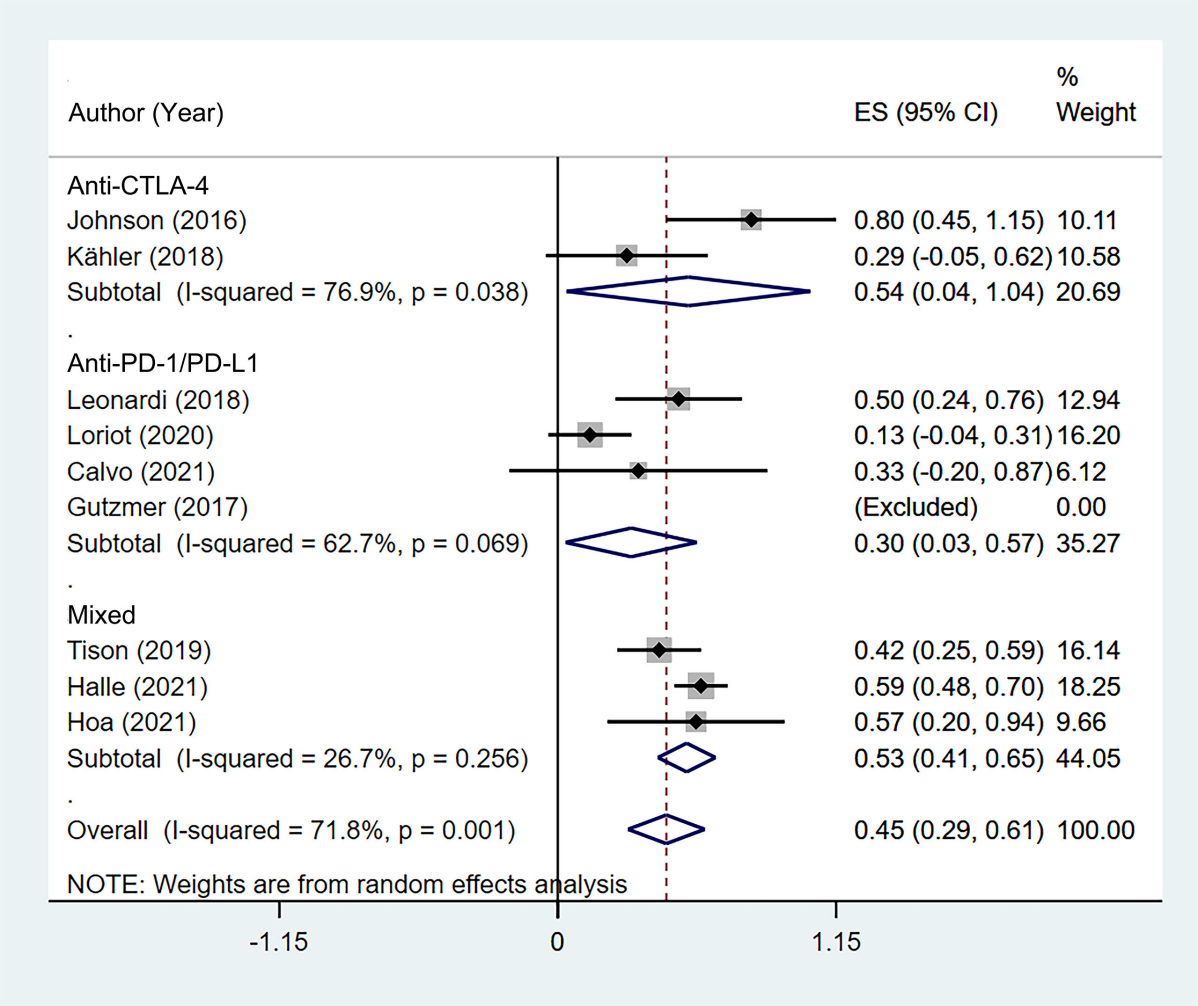
Subgroup analysis of the pooled incidence of *de novo* immune-related adverse event (irAE) in patients with cancer and preexisting psoriasis based on the class of immune checkpoint inhibitors (ICIs).

According to the class of ICIs, the incidence of *de novo* irAEs was 54.0% (95% CI: 3.6%–104.4%) for anti-CTLA-4, 30.3% (95% CI: 3.2%–57.4%) for anti-PD-1/PD-L1, and 53.3% (95% CI: 41.4%–65.2%) for the mixed group (**Figure 5**). There was no difference in incidence between the anti-CTLA-4 group and anti-PD-1/PD-L1 group (χ^2^ = 0.998, p = 0.318).

Of the 170 cases from six studies (11, 14, 16, 19–21) that reported the specific types of *de novo* irAEs, 26 patients had endocrinopathies (including thyroiditis and hypophysitis), 23 suffered colitis, 14 suffered hepatitis, 13 had toxicities excluding psoriasis, and 9 suffered arthralgias excluding PsA. Based on the 12 cases, the median onset time was 63 days, with a range of 21 to 270 days. The pooled incidence of grade 3–4 *de novo* irAEs was 16.6% (95% CI: 10.7%–22.5%, I^2^ = 0, p = 0.615; **Figure 4B**). Most of the patients received steroids and symptomatic therapy. However, the outcomes of *de novo* irAEs have rarely been documented.

### Discontinuation Due to Flare/*De Novo* Immune-related Adverse Event and Rechallenge

**Figure 6:**
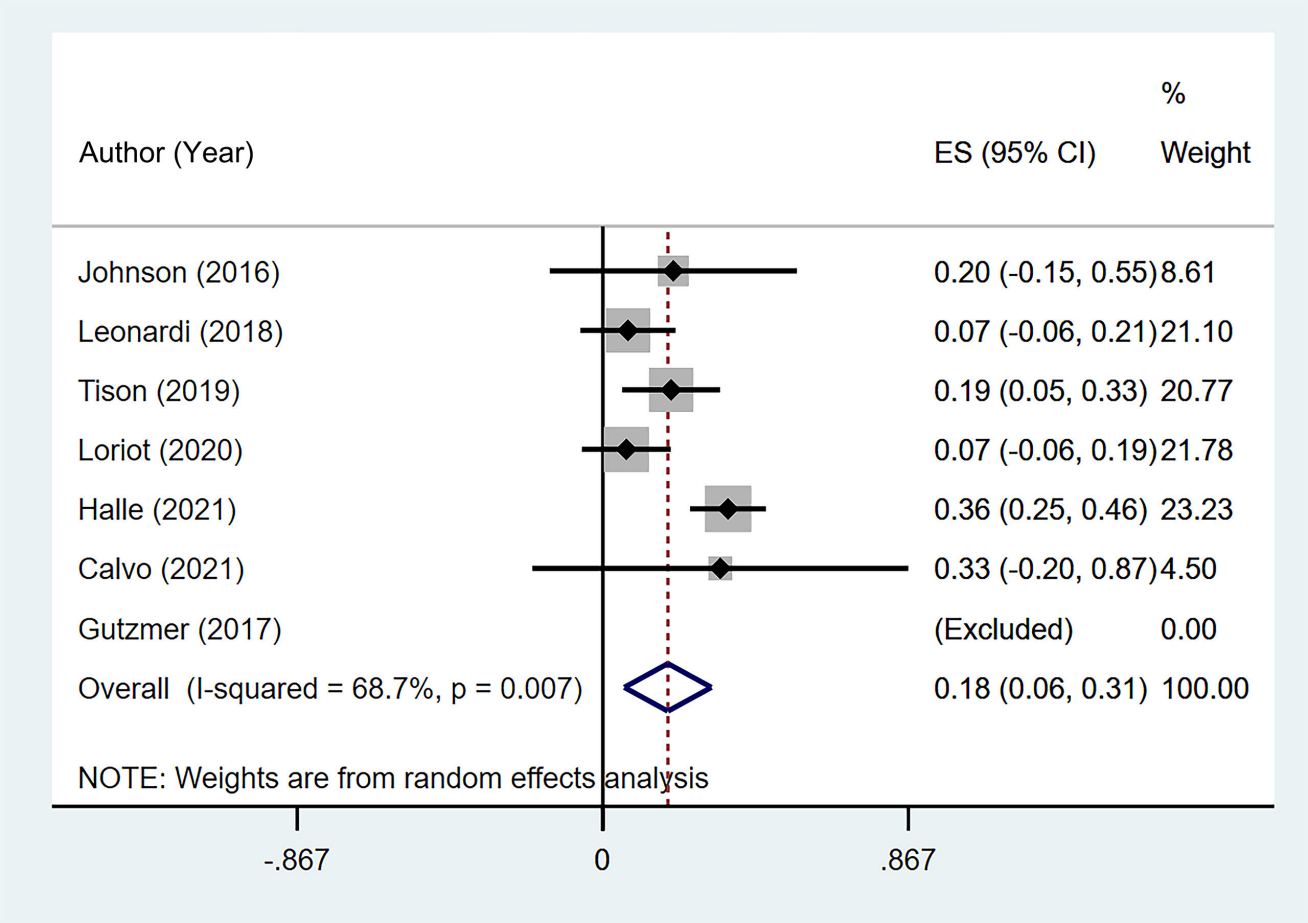
The pooled incidence of immune checkpoint inhibitor (ICI) therapy discontinuation due to flare/*de novo* immune-related adverse event (irAE) in patients with cancer and preexisting psoriasis.

Seven studies (11, 13, 16–20) reported the discontinuation rate of ICI therapy due to flare or *de novo* irAEs. The summary rate was 18.5% (95% CI: 6.1%–30.8%) according to a random-effects analysis. However, significant heterogeneity was noted (I^2^ = 68.7%, p = 0.007; **Figure 6**). One patient with delayed treatment of colitis died.

In three studies (16, 20, 21), twenty-two patients were rechallenged with ICI therapy after flares or *de novo* irAEs. Leonardi et al. (16) reported that a patient suspended anti-PD-1/PD-L1 treatment for about 1 month because of grade 2 thyroiditis, but the symptom persisted after the rechallenge. Twenty patients in the study by Halle et al. (20) received a rechallenge, involving 13 patients with flares and 7 patients with *de novo* irAEs. One of the nine patients who received the same class of ICI flared. One of 11 patients who changed the ICIs class flared. The flare grades were 1–2 without discontinuation. One patient developed grade 3 colitis and grade 2 rash after rechallenge with anti-CTLA-4. Hoa et al. (21) reported one patient who discontinued ICI for 2 weeks due to a flare and then resumed it without any further AEs.

### Clinical Efficacy of Immune Checkpoint Inhibitor Therapy

**Figure 7:**
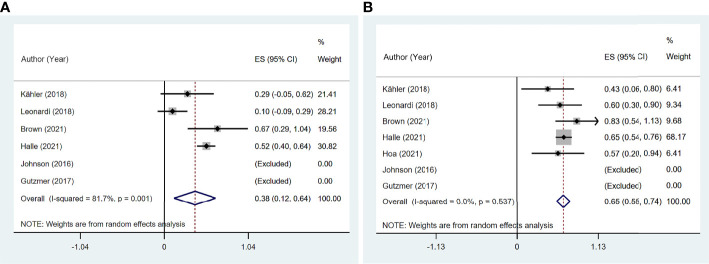
The pooled efficacy in immune checkpoint inhibitor (ICI)-treated patient with preexisting psoriasis. **(A)** Objective response rate (ORR). **(B)** Disease control rate (DCR).

Six studies (11, 13, 14, 16, 19, 20) reported ORR, and seven studies (11, 13, 14, 16, 19–21) reported DCR among 97 patients. The pooled ORR was 38.1% (95% CI: 11.8%–64.3%) with significant heterogeneity (I^2^ = 81.7%, p = 0.001). The summary DCR was 64.5% (95% CI: 55.3%–73.8%), with low heterogeneity (I^2^ = 0, p = 0.537; **Figure 7**).

**Figure 8:**
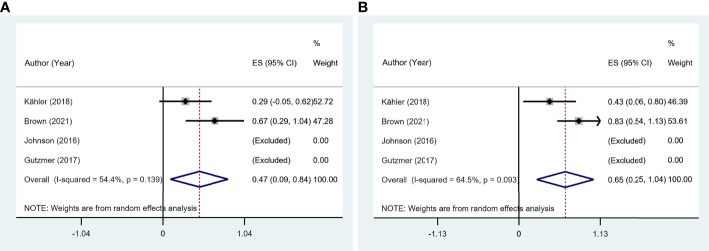
The pooled efficacy in immune checkpoint inhibitor (ICI)-treated patients with preexisting psoriasis and melanoma. **(A)** Objective response rate (ORR). **(B)** Disease control rate (DCR).

Four studies (11, 13, 14, 19) reported 17 patients with preexisting psoriasis and advanced melanoma (**Figure 8**) corresponding to five with CR, two with PR, two with SD, and eight with PD. The pooled ORR was 46.6% (95% CI: 9.3%–83.9%), and the pooled DCR was 64.6% (95% CI: 25.0%–104.1%).

### Risk of Bias Assessment and Publication Bias

**Figure 9:**
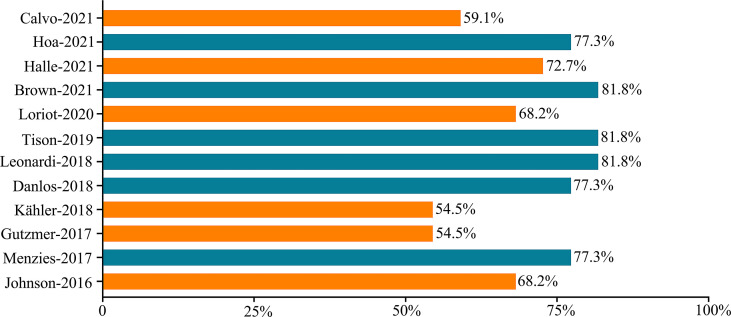
Risk of bias summary.

**Figure 9** shows the risk of bias scored points, which ranged from 12/22 (54.5%) to 18/22 (81.9%) points. Six studies were classified as low risk, while the other six studies were classified as low to medium risk (**Supplementary Table 5**).

**Figure 10:**
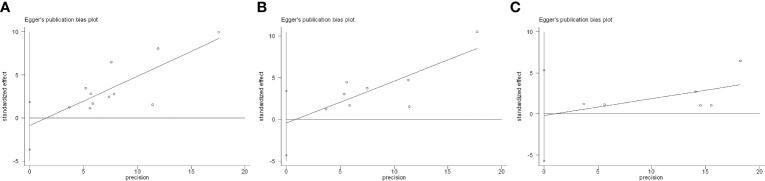
Egger’s test for included studies. **(A)** Flare. **(B)** *De novo* immune-related adverse event (irAE). **(C)** Discontinuation.

There was no evidence of publication bias for safety outcomes based on Egger’s test (all p > 0.05; **Figure 10**).

## Discussion

To the best of our knowledge, this is the first systematic review and meta-analysis to demonstrate the safety, efficacy, and AE management of patients with cancer and preexisting psoriasis treated with ICIs. This study comprehensively indicated a relatively high incidence of flare (45.0%) but a comparable incidence of *de novo* irAE (44.9%) and efficacy of ICIs (ORR, 38.1%; DCR, 64.5%) in those patients. In addition, the majority of AEs were mild and manageable with conventional therapy in our study. The discontinuation rate of flare/*de novo* irAEs was approximately 18.5%.

The summarized incidence of flares in our study was higher than the 35% reported by a previous meta-analysis involving patients with any AIDs (24). Admittedly, psoriasis seems to be more predisposed to flares during ICI therapy. Studies conducted by Kähler et al. (14), Tison et al. (17), and Halle et al. (20) noted that flare occurrence was more frequent in patients with psoriasis and rheumatoid arthritis patients than in other AIDs. ICIs disrupt the CTLA-4 (25) and PD-1/PD-L1 pathways, activate the T cells, and decrease Tregs. They can activate the immune system and promote secondary overexpression of proinflammatory cytokines mediated by Th17 and Th1 lymphocytes (26) with elevated levels of proinflammatory cytokines, such as interleukin-6 (IL-6), interleukin-1β (IL-1β), and tumor necrosis factor-α (TNF-α) (27). Off-target inflammation and autoimmunity exacerbate previous psoriasis (28). However, as co-receptors, the expression and regulation of PD-1 and CTLA-4 differ between numerous AIDs (29). This may explain the high exacerbation rate of psoriasis attributed to the above stronger connections and more mechanisms between the pathways and psoriatic patients than other AIDs.

Most flares were grades 1–2 and could be managed by conventional therapy; only 10.8% of the patients experienced grade 3–4 flares in our data. Different treatments have been used to target skin symptoms as well as extracutaneous issues (such as arthralgia). Most of the patients in our study prioritized topical therapy, with topical corticosteroids and vitamin D analogs being the most commonly used to effectively control the flare, while the severe flares needed systemic therapies and phototherapy. There was no significant preference for immunosuppressants; however, acitretin (30) and apremilast (31) were the most common drugs according to the included studies. Nevertheless, recent strategy guidelines (32) recommended specific selective immunosuppressant drugs, including anti-interleukin-12 (anti-IL-12), interleukin-23 (IL-23), and anti-IL-17 blockade as the priority treatment. We found that systemic steroids were rarely used, owing to earlier concerns that they would intervene with ICI therapy (23), albeit no definitive conclusion has been drawn.

The incidence of ICI therapies varied from 66% to 90% for irAEs of any grade and 14%–43% of grade 3 or higher severity in the general population included in previous clinical trials (33–37). Compared with our results, the incidence (44.9% for any grades) and severity (16.6% for grades 3–4) of *de novo* irAEs did not seem to increase in the targeted population. Concerns about the higher risk of irAEs in patients with preexisting psoriasis have been raised over the few years. However, we now believe it may in part be explained by the lack of distinction between flare and *de novo* irAEs in the past (38). Recent studies (39, 40) also demonstrated that there was no sign of a higher incidence of *de novo* irAEs as compared to the general population.

Moreover, we found that endocrinopathies and colitis were the most common irAEs in patients with preexisting psoriasis. Likewise, Yamaguchi et al. (41) suggested the highest incidence rate (38.3%) of endocrine-related irAEs in patients with a history of AIDs. In other words, endocrine-related syndromes and diarrhea deserve more attention and close monitoring during ICI therapy in those patients. Although the correlation between the class of ICIs and irAE types has been recognized, anti-PD-1 is associated with a high incidence of pneumonia, hypothyroidism, arthralgia, and vitiligo, while anti-CTLA-4 is more inclined to induce colitis, hypophysis, and rashes. Further studies of the relationship between preexisting psoriasis and the subtype of underlying irAEs are warranted. What is more, greater research into AID-related antibodies (Abs) is needed to predict irAEs such as anti-thyroglobulin (Tg) and anti-thyroid peroxidase (TPO) Abs (42, 43).

This study suggested that the median onset was 44 days to flare and 63 days to *de novo* irAEs from the commencement of ICI, which supports the occurrence of flares early during the ICI therapy period. Clarifying the onset of both flare and *de novo* irAEs is crucial for early identification and timely management to minimize the severity. The class of ICIs and types of irAEs that influence the onset time of irAEs have been identified in earlier investigations (44, 45). The skin-related adverse events were regarded to occur the earliest, always within the first two cycles (46), which explains the findings in this study. The findings of Nikolaou et al. (47) supported that patients with prior history of psoriasis suffered from immune-related psoriasis earlier than the general population (5.4 vs. 12.2 months, p < 0.05). Our findings also corroborated the conclusion drawn by Ramos-Casals et al. (48) that most irAEs occur within 2–16 weeks of ICI initiation.

In consideration of the relation between the safety and class of ICIs, for anti-CTLA-4 and anti-PD-1/PD-L1, the pooled incidence of flares was 24.5% and 27.0%, and the pooled incidence of *de novo* irAEs was 54.0% and 30.3%, respectively. There was no significant difference between groups in incidence in our results, although the earlier study found that the type of irAE was different among different ICI classes; anti-CTLA-4 is more susceptible to inducing cutaneous irAE as compared to anti-PD-1/PD-L1 (45).

The fundamental underlying concern is whether the risk of flare and *de novo* irAE and the immunosuppressive treatment for psoriasis, flare, and *de novo* irAE would reduce the efficacy of ICI therapy. The calculated ORR and DCR were 38.1% and 64.5%, respectively, which were comparable to those of previous phase III clinical trials on melanoma (49, 50) and NSCLC (51, 52). As for advanced melanoma, the summarized ORR was 46.6%, and the DCR was 64.6%. There was no significant difference between the ORR and DCR in the earlier trials, which ranged from 17% to 46% and 42% to 63% (53–56). Research on this complex issue has always been controversial, but the majority of studies have indicated that AID patients have at least the same response rate as the general cancer population (57, 58).

In general, we concentrate on psoriasis rather than all AIDs to minimize the heterogeneity across all AIDs and directly guide clinical decision-making. These findings are consistent with the NCCN guidelines and contribute to the current body of evidence.

There are several limitations. First, most of the included patients had clinically inactive diseases. Severe psoriasis had been excluded by clinicians, resulting in an inherent selection bias. Second, significant heterogeneity between the included studies is inevitable for various cancer types, psoriasis subtypes, and classes of ICIs across studies. Third, most included studies were retrospective, and psoriatic patients only comprised a small percentage of included patients; therefore, many crucial parameters such as psoriasis area and severity index (PASI) scores, previous treatment, and the disease activity were not available. Fourth, the sample size of the included population was small, and few studies were included in each subgroup based on ICI class. Hence, the reliability of these results is limited. Moreover, few studies have reported the subsequent management of AEs, such as rechallenge. Finally, few patients may be included in multiple studies. Therefore, these results must be viewed with caution toward clinical application.

## Conclusions

This study revealed that flares are frequent in patients with cancer and preexisting psoriasis treated with ICIs, but with a comparable incidence of *de novo* irAEs and clinical efficacy to the general population. Most AEs are mild and managed with conventional therapies. Patients with preexisting psoriasis should not be deprived of access to ICI therapy. Further prospective trials including those patients are required to provide distinct clinical care guidelines.

## Author Contributions

YY, YZ, and HC designed the study. YY, YZ, and HC screened and selected the records. YY, YZ, XZ, and KT extracted and analyzed the data. YY and JL contributed to the manuscript draft. XZ, JZ, and HC revised the manuscript critically. All authors approved the manuscript version to be published.

## Funding

This study was supported by the National Natural Science Foundation of China (No. 81873396), which was led by HC.

## Conflict of Interest

The authors declare that the research was conducted in the absence of any commercial or financial relationships that could be construed as a potential conflict of interest.

## Publisher’s Note

All claims expressed in this article are solely those of the authors and do not necessarily represent those of their affiliated organizations, or those of the publisher, the editors and the reviewers. Any product that may be evaluated in this article, or claim that may be made by its manufacturer, is not guaranteed or endorsed by the publisher.
