# Supplementary material for: Immune Checkpoint Inhibitors in the Treatment of Patients With Cancer and Preexisting Psoriasis: A Systematic Review and Meta-Analysis of Observational Studies
Source: Front Oncol. 2022 Jul 15;12:934093. doi: 10.3389/fonc.2022.934093 (PMC9334704; doi:10.3389/fonc.2022.934093)
Supplement: Supplementary file 1 [file DataSheet_1.docx]

Supplementary Material

# Appendix 1. Search strategy.

(neoplasm OR neoplasia OR cancer OR malignancy OR malignant OR tumor OR carcinoma OR melanoma OR NSCLC OR leukemia OR lymphoma) AND ("immune checkpoint inhibitor" OR "checkpoint blocker" OR "programmed death receptor 1" OR ICIs OR CTLA-4 OR "cytotoxic T lymphocyte associated protein 4" OR PD-1 OR "programmed death receptor 1" OR "PD-1 Inhibitor" OR PD-L1 OR " programmed death-ligand 1" OR "PD-L1 Inhibitor" OR ipilimumab OR YERVOY OR "MDX CTLA 4" OR "MDX-CTLA-4" OR Yervoy OR "MDX 010" or MDX010 OR BMS-734016 OR tremelimumab OR ticilimumab OR "CP 675" OR "CP675 cpd" OR CP-675 OR "CP 675 206" OR "CP 675206" OR CP-675206 OR nivolumab OR "ONO 4538" OR ONO4538 OR Opdivo OR Nivo OR "BMS 936558" OR BMS936558 OR BMS-936558 OR "MDX 1106" OR MDX1106 OR MDX-1106 OR pembrolizumab OR Keytruda OR Lambrolizumab OR "Merck 3475" OR Merck3475 OR "MK 3475" OR MK3475 OR MK-3475 OR tislelizumab OR BGB-A317 OR "Sch 900475" OR Sch900475 OR camrelizumab OR carrelizumab OR SHR-1210 OR "SHR 1210" OR sintilimab OR IBI308 OR IBI-308 OR "IBI 308" OR toripalimab OR JS001 OR JS-001 OR "JS 001" OR penpulimab OR AK105 OR AK-105 OR "AK 105" OR zimberelimab OR GLS-010 OR GLS010 OR "GLS 010" OR cemiplimab OR REGN2810 OR LIBTAYO OR atezolizumab OR tecentriq OR MPDL3280A OR MPDL-3280A OR RG7446 OR RG-7446 OR durvalumab OR MEDI4736 OR MEDI-4736 OR imfinzi OR avelumab OR bavencio OR MSB-0010682 OR MSB0010682 OR MSB0010718C OR MSB-0010718C OR envafolimab OR KN035 OR sugemalimab OR cejemly) AND (psoriasis OR psoriases OR psoriatic) AND (preexisting OR pre-existing OR history OR prior OR previous OR concomitant OR concurrent OR baseline)

PubMed: 46 results

EMBASE: 226 results

Cochrane library: 7 results

MEDLINE: 41 results

Total: 320 results

# Supplementary Figures and Tables

## Supplementary Tables

### Table 1. Data extraction form

**Data Extraction From**

Article URL:

| First author | Journal/Conference Proceedings | Publication year | Country |
| --- | --- | --- | --- |
|  |  |  |  |

Contact Address:

Source of Sponsorship:

**Study eligibility**

| Item | Observational study | population: with cancer and psoriasis | intervention: treated with anti-PD-1/PD-L1/CTLA-4 inhibitors or combination | Outcomes: safety, efficacy or both |
| --- | --- | --- | --- | --- |
| Choice | Yes / No / Unclear | Yes / No / Unclear | Yes / No / Unclear | Yes / No / Unclear |
| Reason |  |  |  |  |

**Participants and study characteristics**

| Participants characteristics | Further details |
| --- | --- |
| Number of participants were analysed |  |
| Age(mean, median, range, etc) |  |
| Sex of participants (numbers/percentage) |  |
| Psoriasis subtypes |  |
| Autoimmune diseases study? | 1. Yes B. No C. Unclear |
| Study characteristics |  |
| Study design | 1. cohort study B. Case-control study C. Case series |
| Study type | 1. Single-center B. Multi-center C. Prospective D. Retrospective |
| Median (range) length of follow-up days |  |
| Intervention | 1. Anti-PD-1 B. Anti-PD-L1 C. Anti-CTLA-4 D. Combination:_________ |
| The details of intervention |  |

**Outcomes**

| Item | Patients (percentage) | Grade |
| --- | --- | --- |
| Safety |  |  |
| Flare |  |  |
| De novo irAE  Efficacy |  |  |
| CR |  |  |
| PR |  |  |
| SD |  |  |
| PD |  |  |
| CR+PR |  |  |
| CR+PR+SD |  |  |
| Discontinuation |  |  |
| Other |  |  |

irAE, immune-related adverse event; CR, complete remission; PR, partial response; SD, stable disease; PD, progressive disease

### Table 2. Characteristics of the observational studies included in the meta-analysis

| Author-year | Patients(n) | Country | Data source | Period | Study design | Time since psoriasis/ Ps A diagnosis | Median follow-up day | Psoriasis clinical subtype | Type of cancer | Immunotherapy |
| --- | --- | --- | --- | --- | --- | --- | --- | --- | --- | --- |
| Johnson-2016 | 5 | USA | 9 centers | 2012-2015 | Retrospective | 13.5 (0.25-60) | NA | Psoriasis | Melanoma | Anti-CTLA-4 |
| Menzies-2017 | 8 | Australia# | 13 centers | 2012-2015 | Retrospective | NA | 4.7 months | Psoriasis and PsA | Melanoma | Anti-PD-1 |
| Gutzmer-2017 | 3 | Germany | Germany | DeCOG | Retrospective | 7（0.08-28） | NA | Psoriasis vulgaris | Melanoma | Anti-PD-1 |
| Kähler-2018 | 7 | Germany | DeCOG | NA | Retrospective | 9 (0.33-4.92) | NA | Psoriasis and PsA | Melanoma | Anti-CTLA-4 |
| Danlos-2018 | 12 | France | REISAMIC | 2014-2016 | Prospective | 2.5 (0.4-16.1) | 5.1 months | Psoriasis and PsA | Melanoma, NSCLC, etc | Anti-PD-1 |
| Tison-2018 | 31 | France | 3 centers | 2017-2018 | Retrospective | NA | 8 months | Psoriasis and PsA | Melanoma, NSCLC and urologic cancer | Anti-CTLA-4, anti-PD-1, or combination |
| Leonardi-2018 | 14 | USA | 5 centers | 2015-2017 | Retrospective | NA | 17.5 months | Psoriasis and PsA | NSCLC | anti-PD-1 or anti-PD-L1 |
| Loriot-2020 | 15 | France& | 32 countries | 2016-2018 | Prospective | NA | 5.6 months | Psoriasis | Urinary tract  carcinoma | Anti-PD-L1 |
| Brown-2021 | 6 | Australia# | 10 centers | 2015-2020 | Retrospective | NA | 14 months | Psoriasis | Melanoma | Combination of anti-CTLA-4 and anti-PD-1 |
| Halle-2021 | 76 | USA# | 8 centers | NA | Retrospective | NA | 25.1 months | Psoriasis vulgaris, pustular and PsA | Melanoma, NSCLC, head and neck, EAC, etc | Anti-CTLA-4, anti-PD-1, anti-PD-L1 or combination |
| Hoa-2021 | 7 | Canada | 10 centers | 2013-2019 | Retrospective | NA | 11 months | Psoriasis and PsA | Melanoma, lung cancer, etc | anti-PD-1 or combination of anti-PD-1 and anti-CTLA-4 |
| Calvo-2021 | 3 | Spain | 1 center | 2016-2018 | Retrospective | NA | NA | Psoriasis | NSCLC | Anti-PD-1 |

#: Australia, USA and Europe

&: involving 32 countries, Europe, USA, Poland, Brazil, etc.

DeCOG: German Dermatologic Cooperative Oncology Group; REISAMIC: Adverse Events of Immunomodulating Monoclonal Antibodies in Oncology; NSCLC: non-small cell lung cancer; NA: not available.

### Table 3. Meta-regression for the outcome of flare

| Flare | Coef | t | P | 95% CI |
| --- | --- | --- | --- | --- |
| Publication year | 0.051 | 0.040 | 0.205 | -0.028-0.130 |
| Country (US or not) | -0.062 | 0.160 | 0.698 | -0.377-0.252 |
| Study type (prospective or retrospective) | -0.305 | 0.138 | 0.027* | -0.578-0.035 |
| Center (mono or multi) | 0.122 | 0.346 | 0.724 | -0.556-0.799 |
| Sample size (over 20 or not) | 0.222 | 0.149 | 0.135 | 0.250-0.542 |

*p<0.05

### Table 4. Meta-regression for the outcome of *de novo* irAE

| Flare | Coef | t | P | 95% CI |
| --- | --- | --- | --- | --- |
| Publication year | 0.125 | 0.054 | 0.818 | -0.093-0.118 |
| Country (US or not) | 0.371 | 0.145 | 0.010* | 0.088-0.654 |
| Study type (prospective or retrospective) | -0.288 | 0.265 | 0.278 | -0.808-0.232 |
| Center (mono or multi) | 0.057 | 0.383 | 0.882 | -0.694-0.809 |
| Sample size (over 20 or not) | 0.172 | 0.200 | 0.390 | -0.220-0.564 |

*p<0.05

### Table 5. Risk of bias assessment of included observational studies

| Author-year/Term | 1 | 2 | 3 | 4 | 5 | 6 | 7 | 8 | 9 | 10 | 11 | 12 | 13 | 14 | 15 | 16 | 17 | 18 | 19 | 20 | 21 | 22 | Total/Percentage(%) |
| --- | --- | --- | --- | --- | --- | --- | --- | --- | --- | --- | --- | --- | --- | --- | --- | --- | --- | --- | --- | --- | --- | --- | --- |
| Johnson-2016 | 0 | 1 | 1 | 1 | 0 | 1 | 1 | 1 | 0 | 0 | 1 | 0 | 0 | 1 | 1 | 1 | 0 | 1 | 1 | 1 | 1 | 1 | 15/68.2 |
| Menzies-2017 | 0 | 1 | 1 | 1 | 1 | 1 | 1 | 1 | 1 | 0 | 1 | 0 | 0 | 1 | 1 | 1 | 0 | 1 | 1 | 1 | 1 | 1 | 17/77.3 |
| Gutzmer-2017 | 0 | 1 | 1 | 1 | 0 | 1 | 0 | 1 | 0 | 0 | 0 | 0 | 0 | 1 | 1 | 1 | 0 | 1 | 0 | 1 | 1 | 1 | 12/54.5 |
| Kähler-2018 | 0 | 1 | 1 | 1 | 0 | 1 | 0 | 0 | 0 | 0 | 0 | 0 | 0 | 1 | 1 | 1 | 1 | 1 | 0 | 1 | 1 | 1 | 12/54.5 |
| Danlos-2018 | 0 | 1 | 1 | 1 | 1 | 1 | 1 | 1 | 0 | 0 | 1 | 0 | 1 | 1 | 1 | 1 | 0 | 1 | 1 | 1 | 1 | 1 | 17/77.3 |
| Leonardi-2018 | 0 | 1 | 1 | 1 | 1 | 1 | 1 | 0 | 1 | 0 | 1 | 1 | 0 | 1 | 1 | 1 | 1 | 1 | 1 | 1 | 1 | 1 | 18/81.8 |
| Tison-2019 | 1 | 1 | 1 | 1 | 1 | 0 | 1 | 0 | 1 | 0 | 1 | 1 | 1 | 1 | 1 | 1 | 1 | 1 | 1 | 1 | 1 | 0 | 18/81.8 |
| Loriot-2020 | 1 | 1 | 1 | 1 | 1 | 1 | 1 | 0 | 0 | 0 | 0 | 0 | 0 | 1 | 1 | 1 | 1 | 1 | 0 | 1 | 1 | 1 | 15/68.2 |
| Brown-2021 | 0 | 1 | 1 | 1 | 1 | 1 | 1 | 1 | 0 | 0 | 1 | 1 | 0 | 1 | 1 | 1 | 1 | 1 | 1 | 1 | 1 | 1 | 18/81.8 |
| Halle-2021 | 0 | 1 | 1 | 1 | 0 | 1 | 0 | 1 | 0 | 0 | 1 | 1 | 0 | 1 | 1 | 1 | 1 | 1 | 1 | 1 | 1 | 1 | 16/72.7 |
| Hoa-2021 | 1 | 1 | 1 | 1 | 1 | 1 | 0 | 0 | 0 | 0 | 1 | 0 | 1 | 1 | 1 | 1 | 1 | 1 | 1 | 1 | 1 | 1 | 17/77.3 |
| Calvo-2021 | 0 | 1 | 1 | 1 | 1 | 1 | 0 | 0 | 0 | 0 | 0 | 0 | 1 | 1 | 1 | 0 | 0 | 1 | 1 | 1 | 1 | 1 | 13/59.1 |

## 2.2 Supplementary Figures
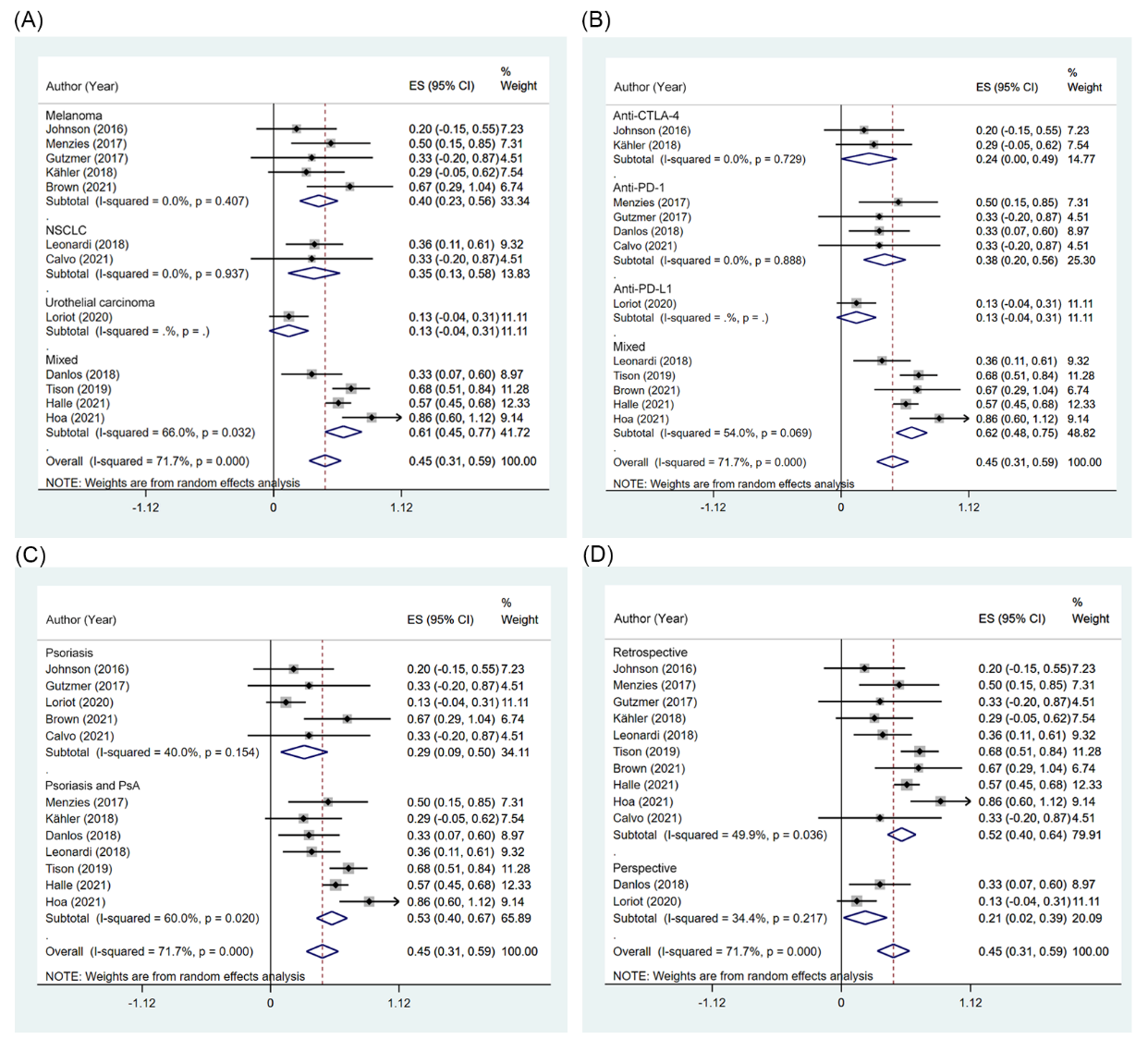


**Supplementary Figure 1.** The pooled incidence of flare according to subtype analysis, stratified by: (A) Cancer types. (B) The class of ICIs [immune checkpoint inhibitors]. (C) Psoriasis subtypes. (D) Study design.


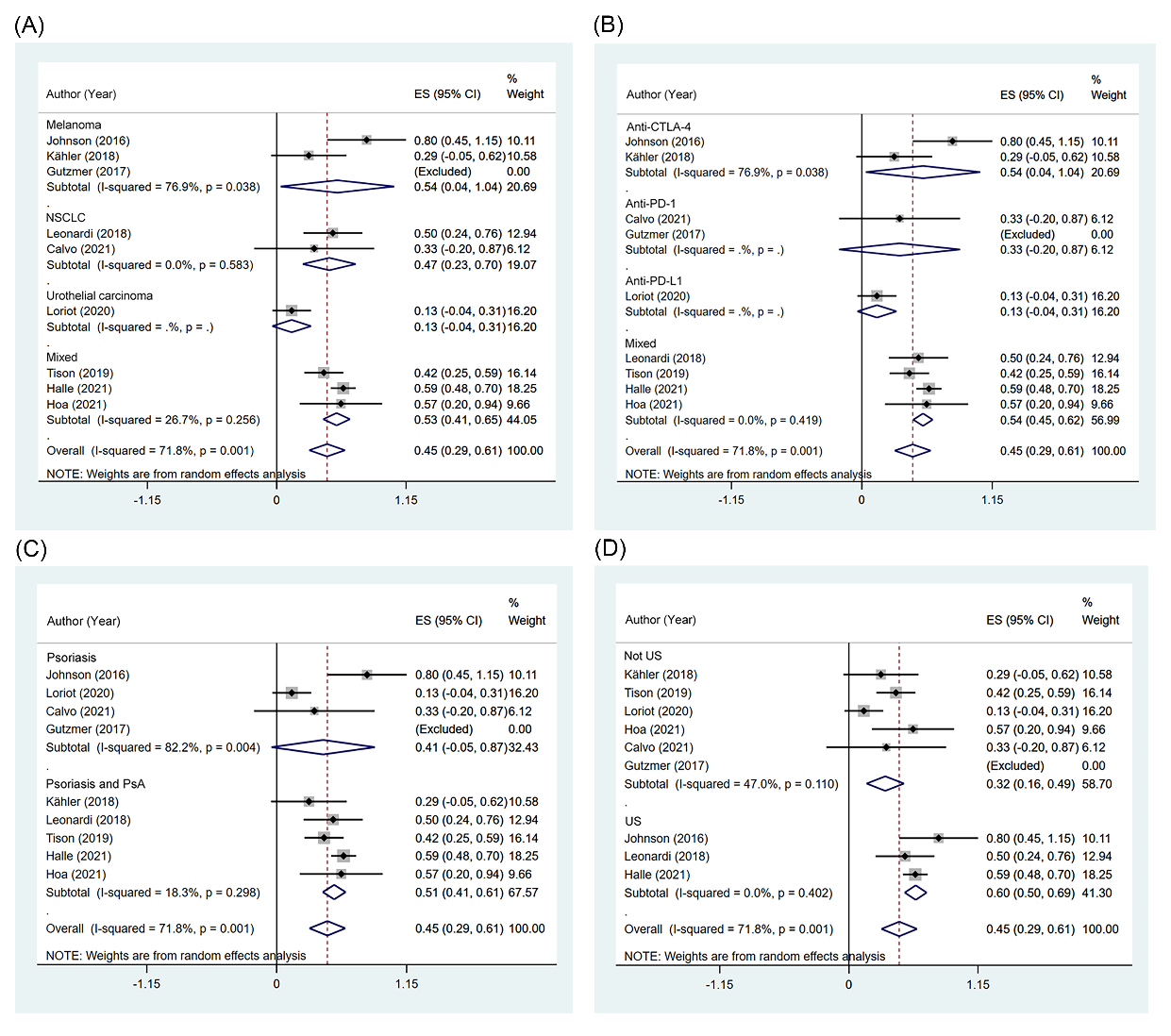


**Supplementary Figure 2.** The pooled incidence of *de novo* irAE [immune-related adverse event] according to subtype analysis, stratified by: (A) Cancer types. (B) The class of ICIs [immune checkpoint inhibitors]. (C) Psoriasis subtypes. (D) Countries.
